# Pregnancy in Liver Cirrhosis: A Rare Clinical Case and Review of Current Management Strategies

**DOI:** 10.3390/jcm15082964

**Published:** 2026-04-14

**Authors:** Nikoleta Stoyanova, Angel Yordanov, Asparuh Nikolov, Zornitsa Gorcheva, Nikola Popovski

**Affiliations:** 1 Department of Obstetrics and Gynaecology, Medical University Pleven, 5800 Pleven, Bulgaria; 2 Department of Gynaecological Oncology, Medical University Pleven, 5800 Pleven, Bulgaria; angel.jordanov@gmail.com; 3 Department of Preclinical and Clinical Sciences, Faculty of Pharmacy, Medical University Pleven, 5800 Pleven, Bulgaria; 4 Department of Nephrology, Haematology and Gastroenterology, Medical University Pleven, 5800 Pleven, Bulgaria; zornica.gorchev@gmail.com

**Keywords:** cirrhosis, pregnancy, disseminated intravascular coagulopathy, miscarriage, intrauterine growth restriction

## Abstract

**Background**: Pregnancy in women with liver cirrhosis is considered a rare clinical condition due to the decreased fertility commonly associated with chronic liver disease. Hormonal disturbances, anovulation and impaired hepatic function contribute to the lower conception rates observed in this population. However, when pregnancy occurs, it is associated with a significantly increased risk of maternal and fetal complications. Maternal risks include hepatic decompensation, variceal bleeding, ascites, coagulopathy and a higher rate of hypertensive disorders during pregnancy and related complications. Fetal complications involve prematurity, intrauterine growth restriction, and increased perinatal mortality. **Methods**: We present the clinical case of a woman with idiopathic liver cirrhosis who experienced four consecutive pregnancies with different clinical courses and outcomes. **Results:** The case highlights the complexity of managing pregnancy in patients with chronic liver disease and underscores the importance of individualized clinical assessment and multidisciplinary management. This report also reviews current management strategies and discusses key considerations for optimizing care in pregnant women with liver cirrhosis. **Conclusions**: Advances in multidisciplinary care and improved management strategies have contributed to better pregnancy outcomes in recent years. Careful monitoring during pregnancy, appropriate management of portal hypertension, and coordinated care between obstetricians, hepatologists, and neonatologists are essential to minimizing potential complications, ensuring favorable maternal and fetal outcomes.

## 1. Introduction

Pregnancy in women with liver cirrhosis is a rare but clinically significant condition, primarily due to reduced fertility associated with chronic liver disease [1]. Nevertheless, the number of reported pregnancies in this population has increased in recent years, largely owing to advances in assisted reproductive technologies and substantial improvements in the management of chronic liver disease and its complications. The estimated prevalence of liver cirrhosis among women of reproductive age is approximately 0.05%, while the incidence of cirrhosis during pregnancy is reported to be around 1 in 4500 pregnancies [2,3].

Globally, chronic viral hepatitis B and C, autoimmune hepatitis, alcohol-related liver disease, and non-alcoholic fatty liver disease (NAFLD) represent the leading causes of liver cirrhosis in women of reproductive age [4].

Pregnancy in women with cirrhosis is associated with an increased risk of both maternal complications and adverse fetal outcomes. Portal hypertension remains the main determinant of maternal and fetal prognosis. Although recent studies have demonstrated a decline in maternal mortality rates, the incidence of maternal and fetal complications remains substantial [4,5].

In a recent meta-analysis, van der Slink et al. reported a maternal mortality rate of 0.89%, most commonly associated with variceal bleeding, with approximately 70% of bleeding episodes occurring during vaginal delivery [1]. Other maternal complications include anemia, postpartum hemorrhage, higher rate of cesarean section, increased rates of puerperal infections, hypertensive disorders of pregnancy, and placental abruption [4,6]. Reported fetal complications include miscarriage, preterm birth, intrauterine growth restriction, and fetal distress [6,7].

Maternal and fetal outcomes largely depend on the severity of the underlying liver disease, with higher complication rates reported in women with advanced cirrhosis, particularly those classified as Child–Pugh class B or C or with elevated MELD scores.

Therefore, pregnancy in women with liver cirrhosis remains challenging and requires preconception counseling, careful multidisciplinary management and individualized risk assessment. Due to the rarity of the condition, current evidence is largely derived from retrospective studies and case series, which makes management decisions particularly challenging.

Herein, we present a rare clinical case of a young woman with idiopathic liver cirrhosis who experienced four consecutive pregnancies with markedly heterogenous clinical courses and outcomes. Notably, the same patient demonstrated a striking variability in pregnancy evolution, ranging from early pregnancy loss complicated by disseminated intravascular coagulopathy (DIC) to an uncomplicated term vaginal delivery without medical follow-up and, subsequently, to a pregnancy complicated by placental insufficiency and intrauterine growth restriction (IUGR) requiring preterm cesarean section.

This intra-individual variability, observed in the context of advanced liver disease, underscores the unpredictable nature of pregnancy outcomes in cirrhotic patients and highlights the limitations of current risk stratification models. To our knowledge, such a spectrum of outcomes across four consecutive pregnancies in a single patient with cirrhosis has not been described.

In addition, we conducted a narrative review of the literature focusing on the etiologies of liver cirrhosis in women of reproductive age as well as the spectrum of maternal and fetal complications, pregnancy outcomes, and modes of delivery. Based on the available evidence, we propose a management algorithm for pregnancy in women with liver cirrhosis, emphasizing the importance of preconception counseling and a multidisciplinary care approach.

## 2. Methodology

To place our case in the context of existing evidence, we performed a review of the literature.

### 2.1. Search Strategy

A narrative literature search was performed across PubMed database using the keywords “liver cirrhosis” AND “pregnancy” AND “case report”. The search was limited to publications in English identified 26 publications between 1994 and 2025.

### 2.2. Inclusion and Exclusion Criteria

To ensure relevance and quality, the following inclusion criteria were applied:

- Peer-reviewed journal articles;
- Studies directly related to pregnancy in women with liver cirrhosis;
- Case report/case series;
- Publications within the defined time frame.

Exclusion criteria included:

- Non-academic sources;
- Studies not directly addressing the research question;
- Duplicate records.

### 2.3. Study Selection Process

**Figure 1:**
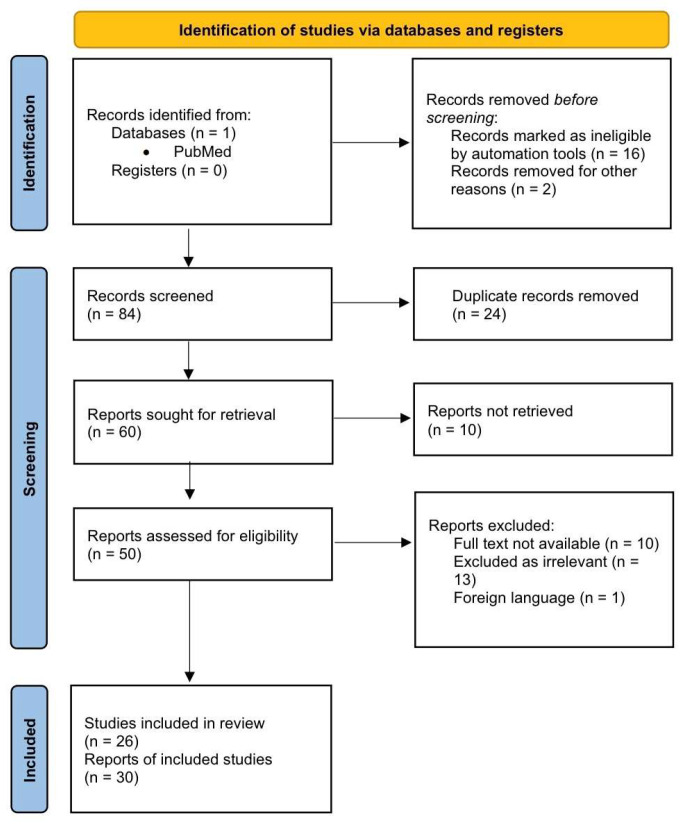
Flowchart illustrates the literature search and selection process for the case reports analyzed in the review and discussion.

The study selection process is summarized in the flowchart on Figure 1. A total of 102 records were initially identified from PubMed. After the removal of 18 records before screening, 84 records remained for screening. Of these, 24 duplicate records were excluded. Subsequently, 60 reports were sought for retrieval, of which 10 were not retrieved. A total of 50 full-text articles were assessed for eligibility. During the full-text review, 24 reports were excluded for the following reasons: lack of full-text availability (*n* = 10), irrelevance to the study topic (*n* = 13), and publications in a foreign language (*n* = 1). Ultimately, 26 studies met the inclusion criteria and were included in the review, comprising a total of 30 reports.

### 2.4. Strength Evidence

The strength of evidence in this study is considered relatively low, as it is primarily based on case reports. While such reports provide valuable clinical insights and detailed observations, they inherently lack generalizability and are subject to a higher risk of bias.

## 3. Case Report

### 3.1. Patient Information

A 23-year-old woman was admitted to the Clinic of Obstetrics and Gynecology in University Hospital Pleven at 13 weeks of gestation in 2021 due to abnormal vaginal bleeding and lower abdominal pain. Her medical history was significant for idiopathic liver cirrhosis diagnosed in 2019, complicated by portal hypertension, ascites, and grade III ruptured esophageal varices, previously managed with Terlipressin. Clinical and laboratory evaluation revealed hepatic encephalopathy; liver disease was classified as Child–Pugh class C.

At the time of initial diagnosis (2019), an etiological workup was performed. Serological testing for hepatitis B virus (HBV) and hepatitis C virus (HCV) was negative. Autoimmune liver disease was considered unlikely based on negative anti-mitochondrial antibodies (AMA), anti-nuclear antibodies (ANA) and smooth muscle antibodies (SMA). The patient denied alcohol consumption or drug abuse. Serum iron and ferritin were within normal ranges, making significant iron overload less likely. Cholestatic liver diseases were considered unlikely, as cholestatic biochemical markers were within normal limits. Serum ceruloplasmin was in normal ranges, therefore Wilson’s disease was excluded.

However, a complete evaluation for other potential causes of cirrhosis was not fully performed. Specifically, testing for α1-antitrypsin deficiency was not available. Additionally, no detailed assessment for vascular causes or rare metabolic liver diseases was conducted.

Liver biopsy was proposed as part of the diagnostic workup; however, the patient declined the procedure.

Given the absence of a clearly identified etiology after the available investigations, the liver disease was classified as idiopathic cirrhosis.

At the time of admission in 2021, standard therapy for the underlying liver condition was indicated but not administered due to the patient’s refusal, despite repeated medical advice.

### 3.2. Pregnancy Complicated by Severe Hemorrhage and DIC (2021)

Physical examination at the time of hospitalization revealed normal pulmonary function and rhythmic cardiac activity. Her blood pressure was 90/60 mmHg, and her heart rate was 90 bpm. No edema of the lower extremities was observed. Gynecological examination revealed a complete abortion, with direct visualization of the amniotic sac and fetus in the vaginal canal. Uterine evacuation by curettage was performed.

During the procedure, the patient developed diffuse uterine bleeding, which persisted postoperatively. Laboratory evaluation demonstrated severe thrombocytopenia, hypofibrinogenemia, elevated D-dimer levels, and prolonged aPTT. Disseminated intravascular coagulation (DIC) was diagnosed and the patient required admission to the intensive care unit for hemodynamic stabilization, transfusion of blood products, and correction of coagulopathy with coagulation factor replacement.

Following clinical stabilization and normalization of laboratory parameters, the patient was discharged. At discharge, she received extensive counseling regarding the importance of regular outpatient follow-up and close surveillance by a gastroenterologist due to her underlying liver cirrhosis and the recent severe complication. Despite these recommendations, she did not attend scheduled visits and was subsequently lost to follow-up.

### 3.3. Subsequent Term Pregnancy Without Follow-Up (2023)

In 2023, the same patient presented with regular uterine contractions at 37 weeks of gestation. It was noted that she had not been receiving regular medical therapy for her liver cirrhosis, had not undergone preconception counseling, and had conceived spontaneously. The pregnancy had not been adequately monitored by either an obstetrician or a gastroenterologist. Physical examination was unremarkable, with no evidence of ascites or hepatic deterioration. Laboratory investigations were within normal limits, except for elevated lactate dehydrogenase (LDH) levels.

She delivered, vaginally, a healthy female neonate weighing 2590 g, with Apgar scores of 8, 9, and 10 at 1, 5, and 10 min, respectively. The postpartum course was uneventful for both mother and newborn.

### 3.4. Pregnancy Complicated by Placental Insufficiency and IUGR (2025)

In 2025, the patient presented at 29 weeks of gestation with complaints of lower abdominal and lower back pain, requiring hospitalization.

Obstetrical examination revealed a tender uterus, with the fundal height at the level of the umbilicus. Fetal movements were reported by the patient. Gynecological examination demonstrated a sacralized, 50% effaced cervix, without dilation. The amniotic sac was intact.

On physical examination, the patient was in good general condition, conscious and oriented, with normal cardiac and pulmonary findings. Blood pressure was 120/70 mmHg, and heart rate was within normal limits. There were no clinical signs of portal hypertension, such as abdominal wall varices or ascites. No peripheral edema or jaundice was observed.

Laboratory findings were within normal limits, except for thrombocytopenia, hypoproteinemia and elevated LDH levels (Table S1).

Indirect cardiotocography showed a normal fetal heart rate with good variability and reactivity, with no signs of fetal distress. Irregular uterine contractions of mild intensity were also observed.

**Figure 2:**
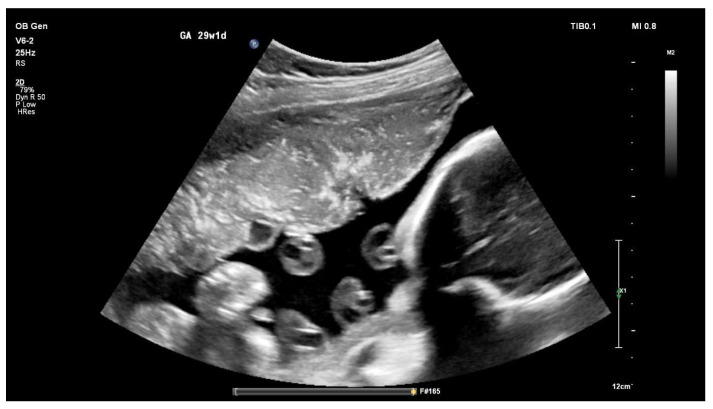
Obstetric ultrasound image showing a heterogeneous placenta with multiple echogenic foci consistent with placental calcifications (advanced placental maturation).

Ultrasound examination revealed intrauterine fetal growth restriction (IUGR) and placental calcifications (Figure 2), raising concern for placental insufficiency in the context of maternal chronic liver disease.

Tocolytic therapy was initiated immediately with a calcium channel blocker and magnesium sulfate in order to prolong pregnancy and allow for fetal maturation. Antenatal corticosteroid therapy was administered for fetal lung maturation, given the risk of preterm delivery.

Fresh frozen plasma and platelet transfusions were administered in the context of thrombocytopenia with the aim of reducing the risk of hemorrhagic complications associated with advanced liver disease and potential preterm delivery.

**Figure 3:**
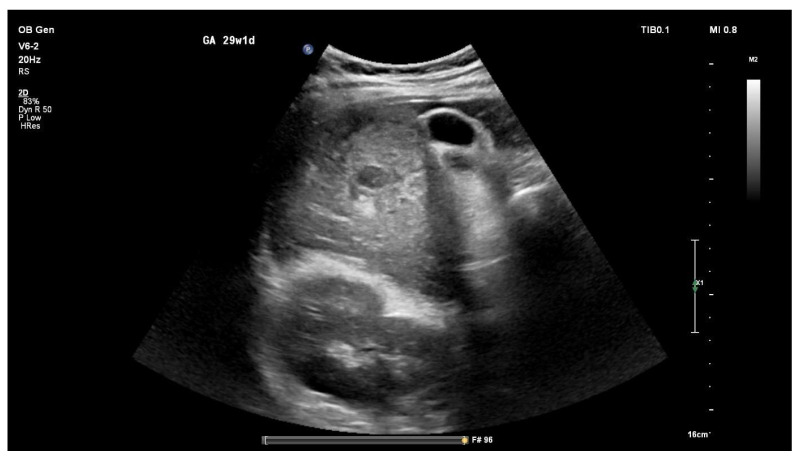
Abdominal ultrasound image demonstrating a cirrhotic liver with irregular, nodular surface contour and coarse, heterogeneous parenchymal echotexture, consistent with advanced chronic liver disease.

A gastroenterology consultation and abdominal ultrasound were performed. No ascites was detected; however, typical sonographic features of liver cirrhosis were present, demonstrated in Figure 3. Two hyperechoic lesions in the right hepatic lobe and subdiaphragmatic region, measuring 57 × 55 mm and 123 × 90 mm, respectively, were identified and suspected to represent hepatic hemangiomas. Splenomegaly with multiple collateral vessels was also noted.

Therapy with Carvedilol was initiated based on the patient’s history of variceal bleeding and the presence of splenomegaly with collateral circulation on ultrasound, suggestive of clinically significant portal hypertension. This approach is consistent with current EASL recommendations for primary prophylaxis of variceal bleeding in pregnant women with cirrhosis (LoE 2, strong recommendation).

The patient was subsequently hospitalized under strict monitoring until the 35th week of gestation. At that time, due to fetal distress in the setting of intrauterine growth restriction (IUGR), a cesarean section was performed at 35 weeks of gestation. The newborn weighed 2100 g and had Apgar scores of 6 and 8 at 1 and 5 min, respectively.

The maternal condition remained hemodynamically stable despite the occurrence of uterine hypotony. Uterotonic agents, including oxytocin and prostaglandins, were administered to promote uterine contraction. Blood transfusion was required during the puerperium due to secondary anemia. Nevertheless, both mother and newborn were discharged 10 days after delivery in good general condition.

### 3.5. Early Pregnancy Termination (2026)

In February 2026, the patient presented with a new pregnancy at 6 weeks of gestation. The pregnancy was terminated by vacuum aspiration without complications. Following the procedure, an intrauterine device was inserted for contraception.

Overall, the patient experienced four pregnancies between 2021 and 2026, with varying clinical course, liver function status, and maternal–fetal outcomes, as summarized in Table 1. Detailed laboratory results are presented in Supplementary Table S1.

## 4. Discussion

### 4.1. Physiological Changes in Pregnancy and Liver Cirrhosis

Pregnancy is characterized by profound hemodynamic, hormonal, and metabolic adaptations that have important implications for women with liver cirrhosis. A hyperdynamic circulatory state develops as early as the second trimester, primarily due to activation of the renin–angiotensin–aldosterone system (RAAS). This leads to increased aldosterone production, sodium and water retention, and a progressive expansion of plasma volume [8]. Cardiac output rises by approximately 50% during the third trimester, accompanied by decreased systemic and splanchnic vascular resistance. In addition, compression of the inferior vena cava by the gravid uterus further alters venous return and intra-abdominal pressure [9].

These physiological changes resemble the hyperdynamic circulation observed in portal hypertension and cirrhosis. Consequently, pregnancy may exacerbate portal hypertension and increase the risk of hepatic decompensation, including ascites formation and rupture in existing esophageal or gastric varices.

Hormonal fluctuations also play a significant role. Elevated levels of progesterone and estrogen influence hepatic metabolism as well as synthetic and excretory liver functions. Transient increases in alkaline phosphatase and alpha-fetoprotein are commonly observed due to placental and fetal production, while serum albumin levels decrease as a result of hemodilution [8,9]. In women with cirrhosis, these physiological laboratory alterations may complicate clinical interpretation and potentially mask early signs of hepatic decompensation.

### 4.2. Clinical Implications of Pregnancy in Women with Liver Cirrhosis

Pregnancy has traditionally been considered a rare event in women with liver cirrhosis for two main reasons. First, advanced liver disease is uncommon during reproductive age. Second, cirrhosis leads to significant hormonal and metabolic disturbances, resulting in anovulation, amenorrhea, and reduced fertility rates [10].

However, advances in early detection of liver dysfunction, the availability of effective pharmacological therapies, and the increasing use of assisted reproductive technologies have made conception and maintenance of pregnancy more achievable. Despite these improvements, pregnancy in women with liver cirrhosis remains a high-risk clinical condition due to the substantial risk of maternal and fetal complications. Moreover, subsequent pregnancies in women with established cirrhosis are particularly uncommon, especially in the absence of adequate control and monitoring of the underlying disease.

### 4.3. Discussion of the Presented Case

The clinical course across the four pregnancies demonstrates the substantial impact of inconsistent disease management and lack of structured follow-up in a young woman with liver cirrhosis.

Furthermore, limitation of the presented case is due to incomplete etiological work-up because of the lack of further investigations in the field of vascular pathologies and alpha-1 antitrypsin deficiency. During the first pregnancy (2021), the absence of regular therapy and monitoring likely contributed to severe decompensation, culminating in miscarriage complicated by disseminated intravascular coagulation (DIC). This presentation highlights the high-risk nature of pregnancy in patients with advanced cirrhosis, particularly without preconception planning and multidisciplinary care.

Despite detailed counseling after discharge, the patient did not engage in follow-up, representing a missed opportunity for disease stabilization and risk assessment. The subsequent pregnancy (2023), although unmonitored and without therapy, resulted in a favorable maternal and neonatal outcome. This underscores the unpredictable course of cirrhosis in pregnancy and may reflect a period of transient hepatic compensation. However, such an outcome should be interpreted cautiously, as it likely represents an exception rather than the norm.

In contrast, the third pregnancy (2025) was complicated by placental insufficiency, intrauterine growth restriction (IUGR), and preterm delivery, findings consistent with chronic maternal disease. The delayed initiation of appropriate therapy further illustrates the consequences of absent longitudinal care, suggesting that earlier intervention and closer surveillance might have mitigated these complications.

Overall, this case emphasizes that the lack of continuous medical therapy, preconception counseling, and regular antenatal follow-up played a central role in shaping both maternal and fetal outcomes. It also highlights the importance of multidisciplinary management and strict surveillance in pregnant patients with advanced liver disease.

A notable feature of this case is the marked heterogeneity of pregnancy outcomes within the same patient, ranging from miscarriage complicated by DIC to successful term delivery and preterm cesarean section due to fetal compromise. This variability suggests that outcomes in women with cirrhosis are not determined solely by the presence of liver disease, but by a dynamic interplay between disease severity, hemostatic balance, and obstetric factors at the time of each pregnancy.

Fluctuations in hepatic function and portal hypertension may partly explain these differences. Even subtle changes can significantly affect coagulation status, predisposing either to hemorrhagic complications, as seen in the miscarriage with DIC, or to relatively stable courses allowing term delivery. Variations in thrombocytopenia and hypersplenism may further influence bleeding risk.

Differences in fetal outcomes may also be linked to variability in uteroplacental perfusion. Chronic liver disease is associated with systemic hemodynamic changes and endothelial dysfunction, which can contribute to placental insufficiency, IUGR, and fetal compromise requiring preterm delivery. Conversely, periods of relative hemodynamic stability may support normal fetal development.

Importantly, the absence of consistent preconception counseling and structured antenatal care likely contributed to the variability observed. Optimized multidisciplinary management may reduce these risks and improve outcomes.

The heterogeneity of outcomes in this patient raises the question of whether such variability is typical in women with liver cirrhosis or represents an exceptional course. To address this, we conducted a literature review of previously reported cases. The main characteristics, complications, and outcomes are summarized in Table 2, with detailed data provided in Table S2.

### 4.4. Etiology of Liver Cirrhosis Among Pregnant Women

Among the included cases, viral (HBV/HCV) and autoimmune etiologies (AIH) accounted for the majority of reported cirrhosis, comprising 16 out of 30 cases (53.3%). Other causes, such as schistosomiasis, alpha-1 antitrypsin deficiency, and congenital vascular abnormalities, were rare and sporadic [14,26,33,36]. These findings reflect the predominance of viral and autoimmune liver disease in women of reproductive age.

Features suggestive of clinically significant portal hypertension, including esophageal varices, ascites, or splenomegaly, were reported in 15 out of 30 pregnancies (50%), indicating that many patients had advanced liver disease at the time of conception.

**Figure 4:**
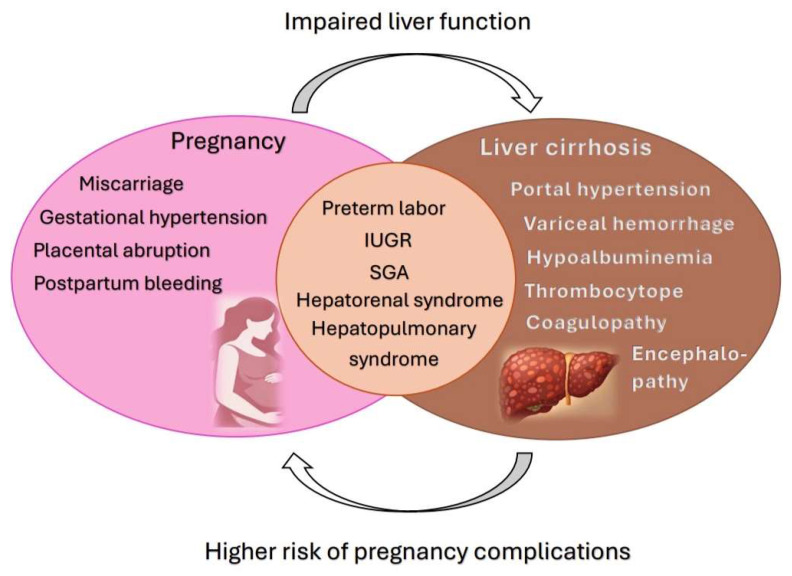
Bidirectional relationship between liver cirrhosis and pregnancy complications.

Pregnancies in women with liver cirrhosis are associated with an increased risk of maternal and fetal complications, including miscarriage, gestational hypertension, preterm birth, and fetal growth restriction [37]. At the same time, physiological changes during pregnancy may further impair liver function and worsen portal hypertension. The bidirectional relationship between liver cirrhosis and pregnancy complications is illustrated in Figure 4.

### 4.5. Maternal Complications

Available evidence suggests that maternal mortality in pregnant women with liver cirrhosis has decreased over time, with reported rates below 2% [8,38]. A recent meta-analysis reported a maternal mortality rate of 0.89%, most commonly due to variceal hemorrhage and hepatic decompensation [1]. Nevertheless, hepatic decompensation remains a significant concern, occurring in up to 25% of pregnancies in women with cirrhosis [4,8]. The risk of deterioration during gestation is closely related to the severity of the underlying liver disease, and a history of pre-pregnancy hepatic decompensation is considered a major predictor of adverse maternal outcomes [4].

The Model for End-Stage Liver Disease (MELD) score has been shown to be a reliable tool for predicting the risk of hepatic decompensation during pregnancy [39]. A recent study reported that a preconception MELD score ≥ 10 demonstrated 83% sensitivity and specificity for predicting liver decompensation during gestation [39,40].

In our review, two maternal deaths were identified, occurring in the context of severe hepatic decompensation, variceal hemorrhage, and acute kidney injury [11,28]. These findings underscore that, although uncommon, maternal mortality is higher than the general population and remains a serious risk, particularly in women with advanced or unstable liver disease.

The metabolic demands of pregnancy, combined with pre-existing impairment of hepatic synthetic and excretory function, may precipitate clinical deterioration, including jaundice, worsening ascites, and hepatic encephalopathy. In addition, cirrhosis is characterized by a fragile hemostatic balance. Coagulopathy and thrombocytopenia, often exacerbated by vitamin K malabsorption and hypersplenism, substantially increase the risk of bleeding complications during pregnancy and delivery.

In this context, variceal bleeding remains one of the most serious and potentially life-threatening complications often occurring in the second trimester or during second stage of labor. It has been reported in up to one third of cases and in nearly half of patients with portal hypertension [41]. In our review, varices were identified in 10 of 30 pregnancies, and one case was complicated by fatal hemorrhage affecting both mother and fetus, underscoring the severity of this condition [11].

Postpartum hemorrhage represents another major bleeding complication in this population, largely attributable to impaired hepatic synthesis of coagulation factors, thrombocytopenia, and hypersplenism. These pathophysiological mechanisms necessitate close laboratory monitoring and proactive peripartum management.

Beyond variceal hemorrhage, other vascular complications related to portal hypertension should also be considered. Splenic artery aneurysm, although rare, occurs more frequently in patients with portal hypertension and carries a particularly high risk of rupture during pregnancy due to increased blood volume, hormonal influences on vascular integrity, and elevated intra-abdominal pressure [42]. Rupture of a splenic artery aneurysm is associated with catastrophic maternal and fetal mortality and should be recognized as a life-threatening but potentially underdiagnosed complication in this population.

Despite the substantial risk profile, the majority of reported cases resulted in favorable maternal outcomes, likely reflecting improved multidisciplinary care, early identification of complications, and timely intervention. These observations highlight the critical importance of coordinated management involving obstetricians, hepatologists, anesthesiologists, and neonatologists.

In our patient, massive hemorrhage complicated by disseminated intravascular coagulation occurred following second-trimester loss in her first pregnancy, requiring ICU admission, blood product transfusion, and correction of coagulopathy. This severe maternal complication highlights the unpredictable course of cirrhosis during pregnancy, particularly in the absence of regular follow-up and preconception counseling. Notably, subsequent pregnancies resulted in favorable maternal outcomes despite the lack of consistent monitoring or control of the underlying liver disease.

### 4.6. Fetal Complications

Fetal outcomes in pregnancies complicated by maternal liver cirrhosis remain variable and are largely influenced by the severity of the underlying disease and the presence of portal hypertension. Preterm delivery was frequently observed in the reviewed cases, with the majority of births occurring between 30 and 36 weeks of gestation. Preterm delivery is often iatrogenic indicated due to maternal deterioration, fetal compromise, or obstetric complications.

Intrauterine growth restriction (IUGR) was also reported in several cases, particularly in women with advanced liver dysfunction or significant portal hypertension. Impaired uteroplacental perfusion, chronic maternal hypoproteinemia, and hemodynamic instability may contribute to placental insufficiency and restricted fetal growth. In our patient’s third pregnancy, intrauterine growth restriction was identified at 29 weeks of gestation. Despite these complications, timely obstetric intervention allowed delivery of a healthy neonate, demonstrating that favorable fetal outcomes are possible even in high-risk maternal settings.

Although most of the reviewed case reports described favorable neonatal outcomes, larger cohort data suggest that livebirth rates in pregnancies complicated by maternal cirrhosis remain variable, ranging between 58% and 100% [38]. Perinatal mortality was documented in a minority of cases, often associated with extreme prematurity or severe maternal decompensation [11,13,19,33]. Neonatal intensive care unit (NICU) admission was required in selected cases due to prematurity-related complications [18].

### 4.7. Management of Pregnancy in Women with Liver Cirrhosis

Management of pregnancy in women with liver cirrhosis requires a multidisciplinary approach involving hepatologists, obstetricians specialized in high-risk pregnancies, anesthesiologists, and neonatologists. Careful assessment of disease severity and close monitoring throughout pregnancy are essential to reduce maternal and fetal complications.

- Pre-conceptional counseling

The European Association for the Study of the Liver recommends that, whenever possible, women with cirrhosis undergo pre-conceptional counseling [43]. As pregnancy outcomes are closely related to the severity of the underlying liver disease, careful risk stratification is essential to assess the likelihood of hepatic decompensation and adverse maternal and fetal outcomes.

The severity of liver disease should be evaluated using established scoring systems such as the Child–Pugh and Model for End-Stage Liver Disease (MELD) scores. A MELD score < 6 is associated with favorable pregnancy outcomes, whereas a MELD score > 10 predicts an increased risk of hepatic decompensation during pregnancy [41].

Gonsalkorala et al. reported that a preconception albumin–bilirubin (ALBI) score ≤ 2.7 strongly predicted live birth. In addition, a preconception aspartate aminotransferase (AST)-to-platelet ratio index (APRI) of 0.84 was associated with pregnancies reaching term (≥37 weeks). In contrast, higher ALBI grades were associated with shorter gestational duration and an increased risk of preterm birth [5].

Patients with a high risk of decompensated cirrhosis, significant portal hypertension, or a history of variceal bleeding should be counseled regarding the increased risk of adverse maternal and fetal outcomes.

Screening for esophageal varices with upper gastrointestinal endoscopy is recommended within one year of conception, particularly in patients with a history of portal hypertension, decompensation or previous bleeding, so that variceal management can be optimised [43].

In addition, optimization of liver function and review of current medications are essential to discontinue potentially teratogenic drugs. Multidisciplinary counseling should be provided to allow informed decision-making regarding pregnancy.

- Evaluation during early pregnancy

Once pregnancy is confirmed, early evaluation should be performed in a tertiary care center with expertise in managing high-risk pregnancies. Baseline laboratory investigations should include liver function tests, coagulation profile, platelet count, and renal function assessment. Imaging studies such as abdominal ultrasound may be useful for evaluating liver morphology and signs of portal hypertension.

Patients should also be assessed for the presence of complications including ascites, hepatic encephalopathy, and variceal disease. If screening endoscopy has not been performed prior to pregnancy, it should preferably be undertaken during the second trimester [43]. Identified varices should be managed appropriately with initiation or optimization of beta-blocker therapy and endoscopic treatment of high-risk varices (large varices or those with red wale signs), typically by endoscopic band ligation. For primary or secondary prophylaxis of variceal bleeding, non-selective beta-blockers may be initiated or continued during pregnancy, as the potential benefits generally outweigh the risks of fetal growth restriction or neonatal hypoglycemia. Propranolol and Carvedilol are classified as FDA category C and, although their possible adverse effects on the fetus, are considered the drugs of choice in pregnant women with liver cirrhosis [8,44]. In a study of women with established cardiovascular disease treated with beta-blockers throughout pregnancy, carvedilol was not associated with fetal growth restriction, suggesting a potential advantage compared with propranolol [45].

- Monitoring during pregnancy

Pregnant women with cirrhosis require regular follow-up throughout gestation. Maternal monitoring typically includes periodic assessment of liver function tests and clinical evaluation for signs of hepatic decompensation. Attention should be paid to complications related to portal hypertension, including variceal bleeding and ascites.

Fetal surveillance is also essential due to the increased risk of intrauterine growth restriction and preterm delivery. Serial ultrasound examinations are recommended to monitor fetal growth and well-being.

- Management of complications

Variceal bleeding remains one of the most serious complications during pregnancy in women with cirrhosis. The general principles for the treatment of acute variceal bleeding apply to pregnant patients with cirrhosis, although certain modifications are required. In the setting of acute hemorrhage, therapy should include vasoactive agents such as octreotide together with broad-spectrum antibiotics [43,46]. Terlipressin is generally avoided during pregnancy because its potent vasoconstrictive effects may provoke uterine contractions, reduce uteroplacental blood flow, and lead to fetal ischemia, potentially resulting in intrauterine demise or placental abruption [47]. Consequently, it should be considered only when both endoscopic therapy and octreotide have failed. Endoscopic variceal ligation (EVL) is regarded as the preferred endoscopic treatment for acute esophageal variceal bleeding [44,48,49]. Injection sclerotherapy has also been used historically; however, the potential passage of sclerosant substances into the placental circulation remains a theoretical concern [50]. In cases of persistent or refractory bleeding despite optimal endoscopic management, placement of a transjugular intrahepatic portosystemic shunt (TIPS) may be considered. TIPS has been successfully used not only to control hemorrhage but also to facilitate cesarean delivery in patients with extensive abdominal wall varices [51]. Nevertheless, the decision to proceed with TIPS during pregnancy should be based on careful multidisciplinary evaluation and a thorough risk–benefit assessment.

Ascites should be managed with sodium restriction and, if necessary, cautious use of diuretics under close monitoring. Hepatic encephalopathy may be treated with lactulose and identification of precipitating factors. In cases of hepatic decompensation, hospitalization and intensive multidisciplinary management are required.

- Delivery planning

Delivery in pregnant women with cirrhosis should ideally take place in a tertiary care center with expertise in managing high-risk pregnancies, where both maternal and neonatal complications can be appropriately addressed.

In general, vaginal delivery is preferred unless obstetric indications necessitate cesarean section [43,49]. Attention should be given to minimizing a prolonged second stage of labor in women with significant portal hypertension, as increased intra-abdominal pressure may theoretically increase the risk of variceal bleeding.

The optimal mode of delivery in women with cirrhosis remains individualized and is primarily determined by obstetric indications and the maternal clinical condition. In reported cases, cesarean delivery has been performed relatively frequently, particularly in the presence of fetal distress, severe portal hypertension, or maternal decompensation. However, several reports have also described successful vaginal deliveries, including induced labor, without major maternal complications.

The relatively high rate of cesarean sections likely reflects concern regarding the potential risk of variceal bleeding during the second stage of labor. Nevertheless, current evidence does not support cirrhosis itself as an absolute indication for operative delivery [38,52]. Careful evaluation of maternal hemodynamic status, coagulation parameters, and the severity of portal hypertension is essential when determining the safest mode of delivery.

In our case, the patient’s second pregnancy resulted in an uncomplicated spontaneous vaginal delivery. During the third pregnancy, cesarean section was performed because of fetal distress rather than maternal hepatic deterioration, further supporting the principle that the mode of delivery should be individualized for each pregnancy.

- Postpartum management

The postpartum period in women with cirrhosis is associated with an increased risk of complications and therefore requires careful monitoring. Postpartum hemorrhage has been reported in approximately 5–45% of pregnancies in women with cirrhosis and is likely related to multiple factors, including coagulopathy, thrombocytopenia, and the presence of ectopic varices [38,52]. Management of postpartum hemorrhage may require a combination of blood product transfusion, uterotonic agents and, when necessary, surgical intervention.

In addition to hemorrhagic complications, the postpartum period may also be associated with hepatic decompensation. For this reason, close clinical and laboratory monitoring is recommended after delivery. Reassessment of the underlying liver disease should be performed, and patients should receive appropriate counseling regarding contraception and planning of future pregnancies.

**Figure 5:**
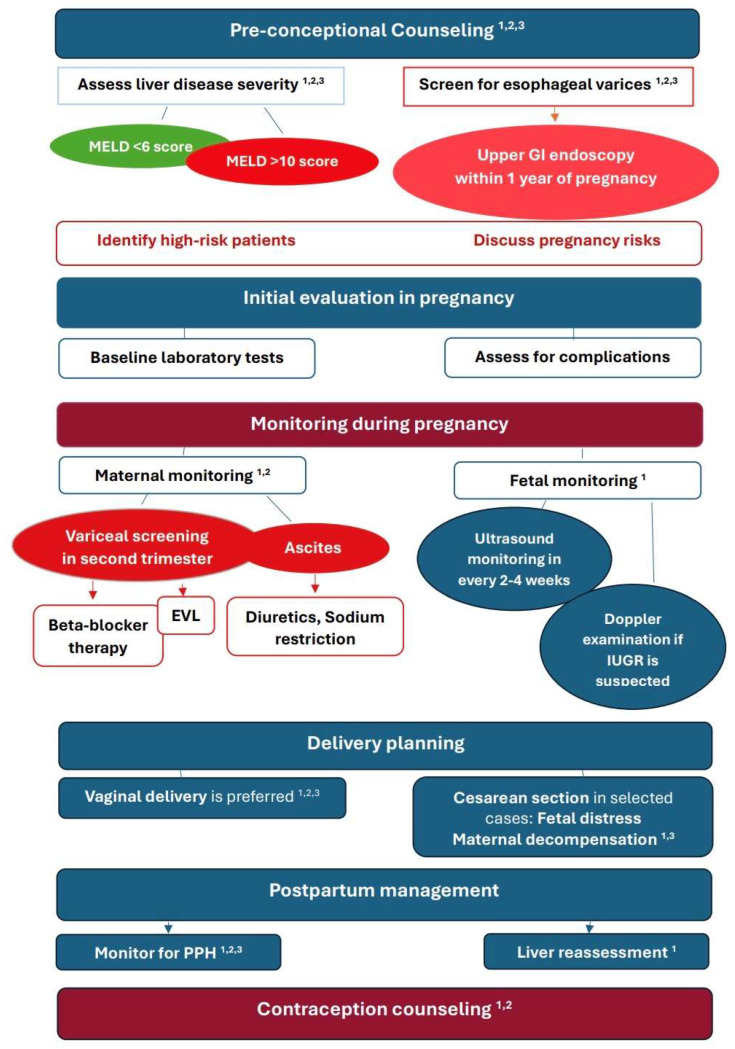
Proposed management algorithm for pregnancy in patients with cirrhosis. Recommendations are based on current international guidelines and evidence discussed in the main text, including: ^1^ EASL Clinical Practice Guidelines on the management of liver diseases in pregnancy [43]; ^2^ AASLD Practice Guidance on Reproductive Health and Liver Disease (Hepatology, 2021 [49]; ^3^ FIGO guideline on liver disease and pregnancy (2025)) [44].

Figure 5 summarizes algorithm for management of pregnancy in women with liver cirrhosis focusing on preconception assessment, risk stratification, and close monitoring throughout pregnancy and postpartum period.

## 5. Conclusions

Our clinical case demonstrates four consecutive pregnancies in a patient with idiopathic liver cirrhosis, each with a different maternal and fetal outcome. A review of 30 published case reports highlights that pregnancies in women with cirrhosis carry significant maternal risks, including variceal bleeding, hepatic decompensation, and postpartum hemorrhage, while fetal complications such as prematurity and growth restriction are also frequent. Despite these risks, favorable maternal and neonatal outcomes are possible with careful surveillance and proactive multidisciplinary management. This case, in the context of the existing literature, underscores the unpredictable course of cirrhosis during pregnancy and emphasizes the importance of preconception counseling, individualized risk assessment, and close collaboration between obstetricians, hepatologists, anesthesiologists, and neonatologists to optimize both maternal and fetal outcomes.

## Figures and Tables

**Table 1:** Clinical characteristics, management, and outcomes of four pregnancies in a patient with liver cirrhosis.

Year	Gestational Age	Liver Status	Portal Hypertension Features	Key Laboratory Results	Complications	Treatment	Delivery	Maternal Outcome	Fetal Outcome
2021	13 weeks	Child-Pugh B MELD 40	History of variceal bleeding	↓ Platelets, ↓ Fibrinogen, ↑ D-dimer, ↑ aPTT	Abortion, severe hemorrhage, DIC	Curettage, ICU admission, transfusions	-	Stabilized, lost to follow-up	-
2023	37 weeks	Child-Pugh A MELD 8	None evident	↑ LDH	None	No therapy	Vaginal	Uneventful	Healthy neonate (2590 g, Apgar 8/9/10)
2025	29–35 weeks	Child-Pugh A MELD 11	Splenomegaly, collaterals	↓ Platelets, ↓ Albumin, ↑ LDH	Placental insufficiency, IUGR, fetal distress	Tocolysis, FFP, Platelets, Carvedilol	Cesarean section	Uterine hypotony, stabilization	Neonate 2100 g, Apgar 6/8
2026	6 weeks	Child Pugh A MELD 8	-	-	-	Vacuum aspiration, IUD	-	Uneventful	-

**Table 2:** Overview of reported cases of liver cirrhosis and pregnancy.

Author, Year	Etiology	Complications	Maternal Outcome	Fetal Outcome
Lozano A. et al., 1997 [11]	Alcoholic	Bleeding from esophageal varices	Exitus	Exitus
Zvárová V. et al., 2021 [12]	NA	None	Successful after TIPS	Successful
Yu Y. et al., 2022 [13]	HBV	Solid pseudopapillary tumor of the pancreas, decompensation of cirrhosis, postpartum bleeding	Successful	Exitus
Sreenisha S S. et al., 2023 [14]	Schistosomiasis	Esophageal varices	Successful after EVL at 18 and 21 week	Successful
Tan YW et al., 2018 [15]	PBC	ICP	Successful	Successful
Goh SK. et al., 2001 [16]	PBC	Portal hypertension, splenic varices	Uneventful	Successful
Lelei-Mailu FJ et al., 2018 [17]	HBV	Portal hypertension, ascites, bilateral pleural effusion	Uneventful	Successful
Paramamanathan CP et al., 2025 [18]	Idiopathic	Late FGR	Uneventful	NICU admission, feeding support
	AIH	Portal hypertension, Esophageal varices, thrombocytopenia	Uneventful	Successful
	HBV	None	Uneventful	Successful
Restaino A. et al., 1996 [19]	NA	Jaundice, portal hypertension, ascites	Uneventful	Exitus on 10th postpartum day due to hemorrhagic interstitial pneumonitis
Roncone E et al., 1994 [20]	Alcoholic	NA	Uneventful	Successful
Mitra S. et al., 2012 [21]	AIH/PBC overlap	None	Uneventful	Hyperbilirubinemia of prematurity
Kouakou F et al., 2012 [22]	HBV	Icterus, ascites	Uneventful	Successful
Subhan A. et al., 2007 [23]	HBV and HCV	Esophageal varices, massive ascites necessitating paracentesis at 28 week	Uneventful	Successful
Rijckborst V. et al., 2018 [24]	NA	None	Uneventful	Successful
Szczepańska M. et al., 2018 [25]	AIH	Esophageal varices, pancytopenia, gestational diabetes	Uneventful	Successful
Alzain FA et al., 2025 [26]	Congenital portal vein stenosis	Generalized body oedema	PPH	Successful
Park C et al., 2020 [27]	Alcoholic	Portal hypertension and esophageal varices leading to TIPS	Uneventful	Successful
Shemies RS. et al., 2024 [28]	AIH	AKI	Exitus 2 days after delivery	Stillborn
	HCV	AKI	Uneventful	Successful
	HCV	Preeclampsia, anasarca	Uneventful	Successful
Bonnin M et al., 2005 [29]	AIH	Encephalopathy	PPH	Successful
Veitsman E et al., 2007 [30]	AIH	HPS	Uneventful	Successful
Oɫdakowska-Jedynak, U et al., 2012 [31]	AIH	None	Uneventful	Successful
Braga A et al., 2016 [32]	AIH	Esophageal varices, splenomegaly, thrombocytopenia	Uneventful	Successful
Robertson M et al., 2017 [33]	α1AT deficiency	Decompensation with jaundice and moderate ascites, preeclampsia	AKI in the postpartum period	Exitus 48 h after delivery
Indirayani, I. et al., 2025 [34]	NA	Esophageal varices IV grade, FGR	Uneventful	Successful
El Bacha et al., 2024 [35]	NA	Esophageal varices, ascites, encephalopathy, FGR	Uneventful	Successful
Galibert, S et al., 2022 [36]	α1AT deficiency, AIH	Esophageal and perigastric varices	Uneventful	Successful

## Data Availability

The original contributions presented in this study are included in the article/Supplementary Material. Further inquiries can be directed to the corresponding author.

## Supplementary Materials

The following supporting information can be downloaded at: https://www.mdpi.com/article/10.3390/jcm15082964/s1, Table S1: Summary of laboratory parameters of the patient during different periods; Table S2: Overview of reported cases of liver cirrhosis and pregnancy.

## Abbreviations

The following abbreviations are used in this manuscript:

TIPS	transjugular intrahepatic portosystemic shunt
EVL	endoscopic variceal ligation
ICP	intrahepatic cholestasis of pregnancy
AIH	autoimmune hepatitis
PBC	primary biliary cholangitis
PPH	postpartum hemorrhage
HBV	hepatitis B virus
HCV	hepatitis C virus
AKI	acute kidney injury
HPS	hepatopulmonary syndrome
α1AT	alpha1-antitrypsin
