# Supplementary material for: Pregnancy in Liver Cirrhosis: A Rare Clinical Case and Review of Current Management Strategies
Source: J Clin Med. 2026 Apr 14;15(8):2964. doi: 10.3390/jcm15082964 (PMC13115964; doi:10.3390/jcm15082964)
Supplement: Supplementary file 1 [file jcm-15-02964-s001.zip › jcm-4241570-supplementary.pdf]

Table S1. Summary of laboratory parameters of the patient during different periods

Parameter	2019 /at the time of decompensation of liver cirrhosis/	2021 /after curettage/	2023 /at 37 week of gestation/	2025 /at 29 week of gestation/	2025 /at 35 week, before SC/	2025 /after SC/	Reference range
**Hemoglobin**	62	90	124	120	117	90	120-160 g/L
**Platelet count**	64	50	143	116	119	110	130-360 x 10^9/L
**INR**	1.22	5.14	1.04	1.19	0.99	0.96	0.9-1.2
**Fibrinogen**	2.67	0.83	4.1	3.8	3.82	3.5	2-4 g/l
**AST**	39	32	24	17	13.5	14	0-40 U/L
**ALT**	29	21	26	14	7.4	7	0-40 U/L
**Total bilirubin**	14.5	18	15	14	18.44	16	0-21 umol/L
**Albumin**	33	21	34	29	35	30	35-52 g/L
**Total protein**	55	43	43	58	63	59	66-87 g/L
**LDH**	366	530	937	671	355	412	240-480 U/L
**Creatinine**	41	62	81	58	55	55	56-115 umol/L
**Urea**	5.8	3.4	3.1	2.9	3.4	3.3	2.8-8.1 mmol/L
**CRP**	39	11	4.6	4	3	3.8	3-5 mg/dL

Table S2. Overview of reported cases of liver cirrhosis and pregnancy

**Author, year**	**Maternal age**	**Etiology**	**Child/MELD**	**Complications**	**Gestational age at delivery**	**Mode of delivery**	**Maternal outcome**	**Fetal outcome**
Lozano A. et al., 1997 [14]	NA	Alcoholic	NA	Bleeding from esophageal varices	NA	NA	Exitus	Exitus

Zvárová V. et al., 2021 [15]	31	NA	NA	None	NA	NA	Successful after TIPS	Successful
Yu Y. et al., 2022 [16]	31	HBV	NA	Solid pseudopapillary tumor of the pancreas, decompensation of cirrhosis, postpartum bleeding	25+6	Vaginal delivery	Successful	Exitus
Sreenisha S S. et al, 2023 [17]	33	Schistosomiasis	NA	Esophageal varices	38	Induction of labour	Successful after EVL at 18 and 21 week	Successful
Tan YW et al., 2018 [18]	32	Biliary cirrhosis	NA	ICP	NA	NA	Successful	Successful
Goh SK. et al., 2001 [19]	39	PBC	NA	Portal hypertension, splenic varices	37	SC	Uneventful	Successful
Lelei-Mailu FJ et al., 2018 [20]	32	HBV	NA	Portal hypertension, ascites, bilateral pleural effusion	36	Vaccum extraction	Uneventful	Successful
Paramamanathan CP et al., 2025 [21]	32	Idiopathic	5(A); 9	Late FGR	36+3	Emergency SC	Uneventful	NICU admission, feeding support
	26	AIH	5(A); 11	Portal hypertension, Esophagea	37+2	Induced	Uneventful	Successful

				l varices, thrombocy topenia		vagina l		
	36	HBV	6(A) ; 6	None	37+3	SC	Uneve ntful	Successful
Restaino A. et al., 1996 [22]	NA	NA	NA	Jaundice, portal hypertensi on, ascites	31	SC	Uneve ntful	Exitus on 10th postpartu m day due to hemorrhagi c interstitial pneumoniti s
Roncone E et al., 1994 [23]	25	Alcoholic	NA	NA	30	SC	Uneve ntful	Successful
Mitra S. et al., 2012 [24]	24	AIH/PBC overlap	-;6	None	35	Vagina l deliver y	Uneve ntful	Hyperbiliru binemia of prematurity
Kouakou F et al., 2012 [25]	24	HBV	NA	Icterus, ascites	35+4	SC	Uneve ntful	Successful
Subhan A. et al., 2007 [26]	32	HBV and HCV	NA	Esophagea l varices, massive ascites necessitati ng paracentes is at 28 week	36	Vagina l deliver y	Uneve ntful	Successful
Rijckborst V. et al., 2018 [27]	29	NA	NA	None	38	Vagina l deliver y	Uneve ntful	Successful
Szczepańs ka M. et al., 2018 [28]	28	AIH	A	Esophagea l varices, pancytope nia,	28+3	SC	Uneve ntful	Successful

				gestational diabetes				
Alzain FA et al., 2025 [29]	23	Congenital portal vein stenosis	NA	Generalized body oedema	36+6	Vaginal delivery	PPH	Successful
Park C et al., 2020 [30]	NA	Alcoholic	NA	Portal hypertension and esophageal varices leading to TIPS	35	SC	Uneventful	Successful
Shemies RS. et al., 2024 [31]	30	AIH	8	AKI	25	Vaginal delivery	Exitus 2 days after delivery	Stillborn
	39	HCV	10	AKI	34	Vaginal delivery	Uneventful	Successful
	38	HCV	9	Preeclampsia, anasarca	34	Vaginal delivery	Uneventful	Successful
Bonnin M et al., 2005 [32]	NA	AIH	NA	Encephalopathy	35	SC	PPH	Successful
Veitsman E et al., 2007 [33]	31	AIH	NA	HPS	37	SC	Uneventful	Successful
Ołdakowska-Jedynak, U et al., 2012 [34]	NA	AIH	NA	None	37	Vaginal delivery	Uneventful	Successful
Braga A, et al., 2016 [35]	NA	AIH	NA	Esophageal varices, splenomegaly,	37	SC	Uneventful	Successful

				thrombocytopenia				
Robertson M, et al., 2017 [36]	22	α1AT deficiency	9	Decompensation with jaundice and moderate ascites, preeclampsia	24	SC	AKI in the postpartum period	Exitus 48 hours after delivery
Indirayani, I. et al., 2025 [37]	22	NA	NA	Esophageal varices IV grade, FGR	35	SC	Uneventful	Successful
El Bacha, et al., 2024 [38]	44	NA	NA	Esophageal varices, ascites, encephalopathy, FGR	34	Vaginal delivery	Uneventful	Successful
Galibert, S et al., 2022 [39]	29	α1AT deficiency, AIH	12	Esophageal and perigastric varices	32	SC	Uneventful	Successful
